# Health-related quality of life and associated factors among health care providers in the northwest of Ethiopia: a multicenter cross-sectional study, 2023

**DOI:** 10.3389/fpubh.2024.1357856

**Published:** 2024-04-02

**Authors:** Fasil Bayafers Tamene, Endalamaw Aschale Mihiretie, Fisseha Nigussie Dagnew, Kale Gubae, Fasika Argaw Tafesse, Samuel Agegnew Wondm

**Affiliations:** ^1^Department of Pharmacy, College of Medicine and Health Science, Debre Markos University, Debre Markos, Ethiopia; ^2^Department of Pharmacy, College of Medicine and Health Science, Bahir Dar University, Bahir Dar, Ethiopia; ^3^Department of Pharmacy, College of Medicine and Health Science, Debre Tabor University, Debre Tabor, Ethiopia; ^4^Department of Pharmacy, College of Medicine and Health Science, Dilla University, Dilla, Ethiopia

**Keywords:** healthcare providers, health related quality of life, World Health Organization quality of life-BREF, Northwest Ethiopia, cross-sectional

## Abstract

**Background:**

The diminished quality of life among healthcare providers (HCPs) could impact both their personal well-being and their ability to effectively fulfill healthcare needs and provide necessary facilities to the public. Furthermore, this decline in quality of life may also significantly influence the overall health of HCPs, regardless of their professional training and duties.

**Objectives:**

The aim of this study was to assess the health-related quality of life (HRQoL) and associated factors among healthcare providers at comprehensive specialized hospitals in the Northwest Ethiopia.

**Method:**

A cross-sectional study was conducted among 412 healthcare providers at comprehensive specialized hospitals in Northwest Ethiopia from June to July 2023. Study participants were enrolled using simple random sampling. Health-related quality of life (HRQoL) was measured using the World Health Organization Quality of Life Scale–Bref Version. Data entry and analysis were performed using Epi-data version 4.6.1 and SPSS version 24, respectively. Binary logistic regression was employed to assess the association between quality of life and independent variables. Variables with a *p*-value <0.05 at a 95% confidence interval were considered statistically significant.

**Result:**

Out of the 422 study participants approached, 412 respondents were included in the final analysis. Poor quality of life was observed in 54.6% of participants. Factors such as working hours per day (AOR = 1.85, 95% CI: 1.12; 3.05), working experience (AOR = 1.95, 95% CI: 1.04; 3.65), and the presence of chronic disease (AOR = 2.11, 95% CI: 1.18; 3.75) were significantly associated with poor quality of life.

**Conclusion:**

This study revealed that more than half of the participants experienced poor quality of life. Specific attention is needed for healthcare providers working for more than 8 h per day, those with less work experience, and those with chronic illnesses in order to improve their quality of life.

## Introduction

Healthcare Providers (HCPs) actively provide essential healthcare services to promote the well-being of individuals and communities, with a focus on disease prevention and overall health maintenance. Many HCPs directly engage in assessing injuries and treating illnesses as part of their commitment to healthcare (1). The entire spectrum of healthcare professionals, including doctors, pharmacists, dentists, nurses, and other allied paramedic staff, collaborates to identify the underlying causes of illnesses. They conduct relevant laboratory tests, implement precise treatment strategies, and offer appropriate counseling to facilitate the prompt recovery of their patients (2). Together, healthcare professionals work to deliver services and resources aimed at ensuring optimal healthcare outcomes for the community (3).

The World Health Organization (WHO) considers quality of life (QoL) as a concept that depends fundamentally on the physical and mental health of an individual, as well as the quality of their social relationships, the grade of their physical and emotional dependency, personal beliefs, and their integration into social groups. For that, the WHO introduced the following dimensions to defineQoL: physical dimension, psychological dimension, level of dependency, social relationships, environment, and spirituality (4). This interpretation underscores the perspective that QoL is subjective, encompassing both positive and negative aspects, and exhibits a multidimensional nature (5).

Globally conducted studies have consistently shown that a decrease in the QoL among healthcare professionals significantly impacts their personal well-being and lives, irrespective of their specific professional roles and responsibilities (6). In addition, the diminished QoL among healthcare professionals may impact their professional capabilities in meeting healthcare requirements and providing necessary facilities to the public (7).

Diminished QoL among healthcare professionals can lead to reduced work capacity, excessive workload, negative emotions, subpar professional performance, and conflicts with colleagues (8). Changes in sociodemographic and socioeconomic factors, heightened professional demands, and advancements in treatment outcomes can all potentially influence the QoL of healthcare providers (8). A multitude of factors negatively affected the QoL of HCP. These may include sociodemographic factors such as being female, marital status and high educational status (9, 10), work related variables like being nurse, work experience, working hours and occupational burnout (11–14) as well as clinical related variables like presence of chronic illness and occupational stress (15, 16).

While extensive research exists in the literature exploring the QoL among healthcare professionals in developed nations, utilizing diverse generic and specific QoL measuring tools, there is a shortage of literature investigating QoL among HCPs in developing countries, including Ethiopia. To the best of our knowledge, there is a lack of previous literature assessing health-related quality of life (HRQoL) among healthcare professionals in these regions. Consequently, this study aimed to assess the level of HRQoL and its associated factors among HCP at comprehensive specialized hospitals in the Northwest of Ethiopia.

## Materials and methods

### Study setting, period, and design

A cross-sectional study was conducted from June to July 2023 in five comprehensive specialized hospitals in Northwest Ethiopia: the University of Gondar Comprehensive Specialized Hospital (UoGCSH), Debre Markos Comprehensive Specialized Hospital (DMCSH), Debre Tabor Comprehensive Specialized Hospital (DTCSH), Tibebe-Ghion Comprehensive Specialized Hospital (TGCSH), and Felege-Hiwot Comprehensive Specialized Hospital (FHCSH).

### Population, inclusion, and exclusion criteria

All HCPsworking at comprehensive specialized hospitals in Northwest Ethiopia comprised the source population. The study population included HCPsat comprehensive specialized hospitals who met the inclusion criteria during the data collection period. Healthcare providersaged 18 years and above who provided informed consent were included in the study, while pregnant HCPsand those unwilling to participate were excluded from the study.

### Sample size determination

The single population proportion formula was utilized to determine the required number of participants for this study (17). Since this study was the first of its kind in Ethiopia, a proportion of 50% was used in the sample size calculation. The formula used is as follows $n=\frac{{\left(Z\alpha /2\right)}^{2}\times p\;\left(1-p\right)}{d^{2}}$ where n is represents the desired sample size, Z is the typical normal distribution set at 1.96 (which corresponds to 95% CI), P represents the prevalence utilized in determining the optimal sample size and W is is the degree of accuracy required (with a marginal error of 0.05) Then computing for *n* = 1.96^2*^0.5 (1–0.5)/0.05^2^, *n* = 384. Considering a 10% non-response rate, the final calculated sample size was 422.

### Sampling technique and procedure

Participants from the hospitals were approached using a simple random sampling technique. To ensure representativeness among the hospitals, the sample size was proportionally allocated to each. The number of healthcare providers in the hospitals was 1,099, 381, 536, 453, and 435 from UOGCSH, DMCSH, TGCSH, FHCSH, and DTCSH, respectively. Consequently, we approached 160, 55, 79, 165, and 63 participants from UOGCSH, DMCSH, TGCSH, FHCSH, and DTCSH, respectively.

### Study variables

Health related quality of life was the main outcome variable. The predictor variables were the sociodemographic characteristics of the participants (sex, age, marital status, and educational status), work related variables (like working hours, experience and CPD) and clinical related variables (substance use and presence of chronic illness).

### Operational definitions

**Health related quality of life**: Quality of life was categorized as either good or poor using the Mean WHOQOL-BREF score. Accordingly, participants with a QOL that was lower than or equal to the mean score were classified as having a poor QOL, while those with a QOL that was higher than the mean were classified as having a good QOL (18).

**Substance use**: using at least one of a specific substance (alcohol, Khat or cigarettes) for nonmedical purposes within the last 3 months, according to the alcohol, smoking, and substance involvement screening tool (ASSIST) (19).

### Data collection instrument, procedures, and data quality control

The structured questionnaire employed in this study was adapted from existing literature sources, with adjustments made to suit the context of the study area and the socio-demographic characteristics of the participants (15, 20). Subsequently, the questionnaire underwent translation into the local Amharic language and was then back-translated into English to ensure consistency. The data collection tool comprised four sections. The first part included socio-demographic characteristics of the study participants, such as age, sex, marital status, educational level, and monthly income. The second section covered job and clinical-related characteristics, including the type of profession, working hours, working experience, continuous professional training, and the presence of chronic illness. The third section involved the current substance use assessment tool. ASSIST was utilized to screen participantsbriefly for the use of psychoactive substances. This tool was developed and validated by the WHO (19).

The fourth section consisted of the HRQoL measuring tool. HRQoL was assessed by utilizing the World Health Organization Quality of Life Scale–Bref Version (WHOQoL-BREF), which is a 26-item self-administered generic questionnaire. The WHOQoL-BREF is a sound, cross-culturally valid assessment of HRQoL, as indicated by its four domains: physical health (7 items), psychological health (6 items), social relationships (3 items), and environmental domain (8 items) (21). In addition two individually scored items concerning the individuals’ impressions of their quality of life (QI) and health (Q2) Each of these items was scored from 1 to 5 on a response scale, which is agreed up on as five-point Likert scale (22). To compare domain scores, the mean score of all items in each domain was multiplied by 4, resulting in a “domain raw score” (which ranged from 4 to 20). This domain’s raw score was translated linearly into a domain score out of 100. The overall HRQOL was defined as the average of the four domain scores (23). WHOQoL–BREF has been used in Ethiopia (24–26).

To ensure data quality, the lead investigator organized a one-day training session at each study site for both data collectors and supervisors. A pretest involving 22 healthcare professionals (approximately 5% of the sample population) at Dessie Comprehensive Specialized Hospital was conducted. The pretest aimed to identify any potential issues with the data collection tool and evaluate the clarity, consistency, and ease of use of the questionnaire. Following the pretest, several adjustments were made, including rectifying typing errors and reorganizing the questionnaires. The internal consistency of ASSIST and WHOQoL–BREF was evaluated, yielding Cronbach’s alpha values of 0.78 and 0.85, respectively, indicating acceptable reliability.

### Data processing and analysis

The collected data underwent cleaning, coding, and entry into Epi Data 4.6.0, with analysis conducted using Statistical Package for Social Studies (SPSS) version 24. Descriptive analysis employed mean with standard deviation (SD), frequency, and percentages to explore data distribution. Bivariable and multivariable binary logistic regression analyses were utilized to identify factors associated with overall quality of life. For each variable, the odds ratio (OR) with a 95% confidence interval was computed, along with the corresponding *p*-value, to assess the strength of association. A significance level of <0.05 was used to determine the significance of the association between the outcome and the predictor variables. Model fitness was evaluated using the Hosmer and Lemeshow test, yielding a value of 0.802. Multicollinearity was assessed, with the maximum Variance Inflation Factor (VIF) found to be less than 5, indicating acceptable levels.

## Results

### Sociodemographic characteristics of patients

From 422 samples approached, 412 eligible healthcare providers participated, yielding a response rate of 97.6%. Most respondents (57.8%) were male, averaging 33.6 years of age (±6.4). The majority (58.7%) were single, and a significant portion (70.9%) held a bachelor’s degree. On average, participants earned 8,013 Birr per month (±2017.7) (Table 1).

**Table 1:** Sociodemographic characteristics among healthcare providers (*n* = 412).

Variables	Categories	Frequency (%)	Mean (±SD)
Sex	Male	238 (57.8)	
	Female	174 (42.2)	
Age	<25	121 (29.4)	33.6 (±6.4)
	26–30	190 (46.1)	
	>30	101 (24.5)	
Marital status	Single	242 (58.7)	
	Married	144 (35.0)	
	Divorced	16 (3.9)	
	Widowed	10 (2.4)	
Educational level	Diploma	53 (12.9)	
	Degree	292 (70.9)	
	Masters and above	67 (16.3)	
Monthly income (in birr)			8,013 (±2017.7)

SD, standard deviation.

### Job, clinical, and substance related characteristics of participants

Regarding employment-related attributes, around 25.5% were nurses, while over half, approximately 56.3%, underwent continuous professional training. The majority, approximately 78.6%, worked more than 8 h daily, and over a quarter, around 28.6%, possessed work experience exceeding 5 years. Chronic illnesses were noted in 17.5% of respondents. Substance usage was reported by over a quarter, about 28.2%, predominantly alcohol (73.3%) (Table 2).

**Table 2:** Job, clinical and substance related variables among healthcare providers (*n* = 412).

Variables	Categories	Frequency (%)
Type of profession	Physician	43 (10.4)
	Pharmacist	95 (23.1)
	Nurse	105 (25.5)
	Midwifery	60 (14.6)
	Medical laboratory	42 (10.2)
	Anesthesia	13 (3.2)
	Psychiatrist	17 (4.1)
	Optometrist	10 (2.4)
	Physiotherapist	7 (1.7)
	Public health officer	20 (4.9)
Continuous professional training	Yes	232 (56.3)
	No	180 (43.7)
Working hour (per day)	>8 h	324 (78.6)
	< 8 h	88 (21.4)
Work experience	< 2 years	93 (22.6)
	2–5 years	201 (48.8)
	> 5 years	118 (28.6)
Chronic disease	Yes	72 (17.5)
	No	340 (82.5)
Substance use	Yes	116 (28.2)
	No	296 (71.8)
Types of substance	Alcohol	85 (73.3)
	Cigarette	12 (10.3)
	Khat	19 (16.4)

### Self-rated perceived quality of life and health satisfaction of participants

Around 40.5% of participants indicated their perceived HRQoL as poor. In terms of perceived health satisfaction, over a quarter (26.7%) expressed dissatisfaction, while almost the same proportion (28.7%) reported satisfaction (Table 3).

**Table 3:** Self-rated perceived quality of life and health satisfaction among healthcare providers (*n* = 412).

Variable	Category	Frequency (%)
Perceived quality of life	Very poor	55 (13.4)
	Poor	167 (40.5)
	Neither poor nor good	50 (12.1)
	Good	112 (27.2)
	Very good	28 (6.8)
Perceived health satisfaction	Very dissatisfied	63 (15.3)
	dissatisfied	110 (26.7)
	Neither satisfied nor dissatisfied	71 (17.2)
	Satisfied	118 (28.7)
	Very satisfied	50 (12.1)

### Health-related quality of life of participants

In this study, over half (54.6%) of the participants scored lower than the average in their overall HRQoL. Across the four domains of quality of life, respondents recorded the lowest mean in the psychological domain (44.93 ± 19.38) and the highest mean in the social relationship domain (52.27 ± 25.84). The lowest and highest mean scores in overall HRQOL among the study participants were 22 and 83, respectively (Table 4).

**Table 4:** Mean score of quality of life among healthcare providers (*n* = 412).

Domains	Mean ± SD	95% CI	Range		% of participants who scored below the mean
			Minimum	Maximum	
Physical	48.29 ± 20.82	(46.25–50.27)	19	88	52.7%
Psychological	44.93 ± 19.38	(42.90, 46.67)	13	94	60.7%
Social relationship	52.27 ± 25.84	(49.69, 54.74)	19	94	45.6%
Environmental	45.64 ± 20.92	(43.74, 47.83)	13	88	59.5%
Overall HRQoL	47.78 ± 16.24	(46.18, 49.35)	22	83	54.6%

HRQOL, heath related quality of life; SD, standard deviation; CI, confidence interval.

### Factors associated with overall health related quality of life

Following the analysis using multiple logistic regression, several variables were identified as significantly correlated with HRQoL. Accordingly, healthcare providers (HCPs) who worked over 8 h per day were 1.85 times more prone to having poor HRQoL compared to those working fewer hours (AOR = 1.85, 95% CI: 1.12; 3.05). Similarly, HCPs with less than 2 years of work experience were 1.95 times more likely to report poor HRQoL than those with over 5 years of experience (AOR = 1.95, 95% CI: 1.04; 3.65). Additionally, healthcare providers with chronic illnesses were 2.11 times more likely to have poor HRQoL compared to those without such conditions (AOR = 2.11, 95% CI: 1.18; 3.75) (Table 5).

**Table 5:** Bivariable and multivariable logistic regression and associated factors among healthcare providers (*n* = 412).

Variables	Categories	HRQoL		95% CI		*p* –value
		Good QoL	PoorQoL	COR	AOR	
Sex	Male	117	121	1.62 (1.09–2.41)	1.13 (0.63–2.03)	0.669
	Female	68	106	1	1	
Age	>30 years	49	52	0.62 (0.36–1.07)	0.79 (0.39–1.62)	0.532
	26–30 years	93	97	0.61 (0.38–0.98)	0.74 (0.38–1.41)	0.365
	<25 years	45	76	1	1	
Marital status	Unmarried	114	154	1.38 (0.92–2.08)	1.35 (0.84–2.16)	0.212
	Married	73	71	1	1	
Educational level	Diploma	21	32	1.87 (0.90–3.91)	1.46 (0.61–3.48)	0.387
	Degree	129	163	1.55 (0.91–2.65)	1.68 (0.90–3.14)	0.101
	Masters and above	37	30		1	
Continuous professional training	Yes	96	136	1.44 (0.97–2.14)	1.43 (0.92–2.23)	0.112
	No	91	89		1	
Working hour	>8 h	138	186	1.69 (1.05–2.77)	1.85 (1.12–3.05) *	**0.016**
	< 8 h	49	39		1	
Work experience	<2 years	33	60	2.30 (1.31–4.03)	1.95 (1.04–3.65) *	**0.037**
	2–5 years	88	113	1.63 (1.03–2.57)	1.57 (0.95–2.59)	0.076
	> 5 years	66	52		1	
Chronic disease	Yes	24	48	1.84 (1.08–3.14)	2.11 (1.18–3.75) *	**0.011**
	No	163	177		1	
Substance use	Yes	63	53	1.64 (1.07–2.54)	1.49 (0.91–2.44)	0.108
	No	124	172		1	

*p < 0.05, AOR, adjusted odds ratio, CI, confidence interval; COR, crude odds ratio, bold figures; statistically significant variables.

## Discussion

The demanding and stressful nature of healthcare professions poses a potential threat and can significantly influence the HRQoL of healthcare professionals. Moreover, the particular work environment and interpersonal dynamics can also have an impact on the HRQoL of medical personnel (27). The current study revealed that 54.6% of the study participants scored below the mean score of WHOQOL-BREF, indicating a poor quality of life. Factors such as working hours per day (AOR = 1.85, 95% CI: 1.12; 3.05), working experience (AOR = 1.95, 95% CI: 1.04; 3.65), and the presence of chronic disease (AOR = 2.11, 95% CI: 1.18; 3.75) were found to be significantly associated with poor quality of life.

In this study, 54.6% (95% CI: 49.8; 59.7) of respondents were found to have a poor quality of life, which is consistent with previous studies (28, 29). However, this finding was higher than a study conducted in India (45%) (30) and Brazil (15.4%) (31). On the contrary, the current study reported a lower percentage compared to studies conducted in China (74.6%) (32) and Saudi Arabia (77.5%) (33). The disparities in HRQoL levels could be ascribed to variations in the socio-demographic and economic profiles of the study participants. Furthermore, discrepancies in sampling methods, sample sizes, and criteria for inclusion/exclusion might have contributed to the observed difference.

In this study, healthcare professionals (HCPs) working for more than 8 hours per day were found to have a lower quality of life compared to those working fewer hours. This observation aligns with findings from a study conducted in China (13). The potential explanation for this association could be attributed to the demanding nature of HCPs’ work, characterized by heavy workloads, frequent night shifts, and extended periods of patient interaction. The limited control over work schedules, coupled with the significant patient load, is positively correlated with poorer health and directly contributes to a diminished quality of life. Consequently, it is recommended that hospitals develop scientifically and reasonably structured work schedules to enhance work efficiency and alleviate the workload burden on healthcare professionals.

Healthcare professionals with less than 2 years of work experience were 1.95 times more likely to experience poor health-related quality of life (HRQoL) compared to those with more than 5 years of experience. This can be explained by the notion that less experienced healthcare providers may have a limited understanding of their social, psychological, and environmental needs, leading to a less satisfying life. Additionally, individuals with less experience, particularly younger healthcare professionals, may perceive their lives as more challenging, contributing to a less satisfactory lifestyle (20).

Regarding the presence of chronic disease, healthcare professionals with chronic illnesses exhibited lower quality of life compared to those without chronic conditions. This discovery aligns with studies conducted in Iran (15, 34) and Malaysia (35). The rationale behind this observation is that chronic medical conditions adversely affect quality of life by causing physical impairments, such as increased pain, loss of balance, and reduced muscular strength (36). Consequently, these findings suggest that stakeholders and hospitals should prioritize enhanced healthcare services for their professionals to improve health-related quality of life (HRQoL).

### Strength and limitation of study

This study, the first of its kind in Ethiopia, aimed to assess the level of health-related quality of life and its predictors. However, the findings relied on self-reported data, potentially influenced by participants’ honesty and subject to recall bias. Additionally, assessing substance use may be affected by social desirability bias. The study only included healthcare professionals from governmental health institutions, potentially limiting the generalizability of the findings. Psychological variables were not evaluated in the study. The cross-sectional design precluded follow-up and hindered the establishment of causal relationships. Furthermore, while the WHO BREF assessment tool is cross-culturally valid, its validation in the Ethiopian context has not been established.

## Conclusion and recommendation

This study found that over half of the participants experienced a reduced quality of life. Hence, by developing evidence-based policies and implementing programs focused on improving HRQoL, enhancements are achievable. A scientifically grounded and justified operational plan should be devised and implemented in practical settings to regulate working hours effectively. Regular professional training should be implemented to enhance work experience and skill development. Subsequent research could involve a comparator group, offering a clearer understanding of the impact on healthcare providers’ HRQoL.

## Data availability statement

The raw data supporting the conclusions of this article will be made available by the authors, without undue reservation.

## Ethics statement

The studies involving humans were approved by the Debre Tabor University Research and Publication Directorate, with a reference number of RP/278/23. The studies were conducted in accordance with the local legislation and institutional requirements. The participants provided their written informed consent to participate in this study.

## Author contributions

FBT: Conceptualization, Data curation, Formal analysis, Investigation, Methodology, Software, Supervision, Writing – original draft. EM: Project administration, Supervision, Validation, Writing – review & editing. FD: Resources, Visualization, Writing – review & editing. KG: Methodology, Project administration, Writing – review & editing. FAT: Formal analysis, Software, Writing – review & editing. SW: Data curation, Investigation, Project administration, Resources, Visualization, Writing – review & editing.
